# A Spider Toxin Exemplifies the Promises and Pitfalls of Cell-Free Protein Production for Venom Biodiscovery

**DOI:** 10.3390/toxins13080575

**Published:** 2021-08-18

**Authors:** Tim Lüddecke, Anne Paas, Lea Talmann, Kim N. Kirchhoff, Björn M. von Reumont, André Billion, Thomas Timm, Günter Lochnit, Andreas Vilcinskas

**Affiliations:** 1 Branch for Bioresources, Fraunhofer Institute for Molecular Biology and Applied Ecology, Ohlebergsweg 12, 35392 Giessen, Germany; anne.paas@ime.fraunhofer.de (A.P.); kirchhoff@integrative-venomics.com (K.N.K.); andre.billion@ime.fraunhofer.de (A.B.); andreas.vilcinskas@ime.fraunhofer.de (A.V.); 2 LOEWE Centre for Translational Biodiversity Genomics (LOEWE-TBG), Senckenberganlage 25, 30325 Frankfurt am Main, Germany; bmvr@reumont.net; 3 Syngenta Crop Protection, Werk Stein, Schaffhauserstrasse, CH4332 Stein, Switzerland; lea.talmann@syngenta.com; 4 Institute for Insect Biotechnology, Justus Liebig University of Giessen, Heinrich-Buff-Ring 26-32, 35392 Giessen, Germany; 5 Institute of Biochemistry, Justus Liebig University of Giessen, Friedrichstr. 24, 35392 Giessen, Germany; thomas.timm@biochemie.med.uni-giessen.de (T.T.); Guenter.Lochnit@biochemie.med.uni-giessen.de (G.L.)

**Keywords:** neglected venomous arthropods, spider venom, knottin, biotechnology, synthetic biology, cell-free expression

## Abstract

Arthropod venoms offer a promising resource for the discovery of novel bioactive peptides and proteins, but the limited size of most species translates into minuscule venom yields. Bioactivity studies based on traditional fractionation are therefore challenging, so alternative strategies are needed. Cell-free synthesis based on synthetic gene fragments is one of the most promising emerging technologies, theoretically allowing the rapid, laboratory-scale production of specific venom components, but this approach has yet to be applied in venom biodiscovery. Here, we tested the ability of three commercially available cell-free protein expression systems to produce venom components from small arthropods, using U_2_-sicaritoxin-Sdo1a from the six-eyed sand spider *Hexophtalma dolichocephala* as a case study. We found that only one of the systems was able to produce an active product in low amounts, as demonstrated by SDS-PAGE, mass spectrometry, and bioactivity screening on murine neuroblasts. We discuss our findings in relation to the promises and limitations of cell-free synthesis for venom biodiscovery programs in smaller invertebrates.

## 1. Introduction

Venoms are complex cocktails of bioactive molecules including multiple peptide and protein toxins [1]. Many such toxins have evolved over millions of years under natural selection to fulfil specific biological functions [2]. This process often results in an astonishing degree of target specificity and potency. The bioactivity of toxins makes them promising candidates for the development of novel therapeutics or research tools [3,4,5]. Arthropods provide a rich source of venom components because arthropod venom is extremely complex, including peptides and proteins with useful agrochemical and pharmaceutical applications [6].

Arthropod venom research is hampered by the small size of most species [7]. Venom biodiscovery programs generally require large sample volumes for fractionation, which is difficult to achieve when the target species are small [8,9]. In some cases, hundreds of specimens may be required to accumulate sufficient amounts of venom, which is all but impossible for the smallest arthropods [10]. More recently, venom research has been transformed by innovations in transcriptomics and proteomics. The high sensitivity of mass spectrometry platforms coupled with the inexpensive and rapid sequencing of venom gland transcriptomes allows the analysis of venom components in small sample volumes. This “modern venomics” workflow has enabled the study of venoms from previously neglected arthropods and other invertebrates [11]. However, venomics requires the destruction of sample material, and corresponding bioactivity analysis is usually not possible.

The loss of sample material can be overcome by using a synthetic biology approach to prepare pure toxin molecules. Peptides can be prepared by solid-phase or liquid-phase synthesis or recombinant peptides and proteins can be expressed in prokaryotic or eukaryotic cells [12,13,14,15,16]. One of the most promising developments in synthetic biology is the rise of cell-free protein synthesis systems [17,18]. This involves the use of cytoplasm derived from cell lysates combined with synthetic gene fragments encoding a peptide or protein of interest driven by a promotor compatible with the lysate, allowing the synthesis of any polypeptide. Because the cell-free system is not alive, the efficiency of synthesis is not affected by toxic products. Several cell-free synthesis platforms are commercially available, all allowing the completion of experiments within a few hours [17,19,20]. Theoretically, these cell-free technologies should facilitate arthropod venom biodiscovery because venomics sequence data could be used to generate synthetic gene constructs that would produce sufficient amounts of toxin for laboratory-scale bioactivity studies, thereby circumventing the barrier imposed by limited venom availability. However, these technologies have rarely been explored for their potential to produce venom toxins.

Here, we assessed three commercial cell-free synthesis platforms for their ability to produce the knottin U_2_-sicaritoxin-Sdo1a (USCTX) from the six-eyed sand spider *Hexophtalma dolichocephala* as a model arthropod venom component. This toxin was identified by transcriptome-guided venomics and was structurally characterized by NMR spectroscopy following its expression in heterologous cells [21]. USCTX contains an inhibitory cysteine knot (ICK) motif with complex disulfide bridges, which is an added challenge for in vitro expression [21]. The recombinant USCTX produced by cell-free synthesis was also analyzed to determine its bioactivity. We discuss our findings in terms of the expectations and limitations of cell-free synthesis technologies for future venom biodiscovery programs.

## 2. Results and Discussion

### 2.1. Architecture of the Gene Construct

To assess the ability of different cell-free systems to produce USCTX, we first designed a template from which the genetic machinery within each system could synthesize the mature protein. Although plasmids or linear gene constructs can be used [18], the latter are less expensive to produce and can be generated using standard PCR protocols and/or direct oligonucleotide synthesis.

**Figure 1:**
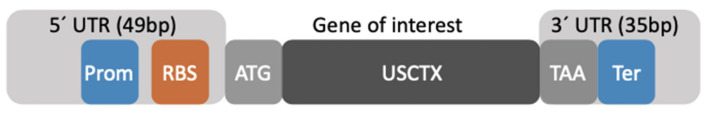
Architecture of the linear gene construct F120. The construct comprises a 49-bp 5′ UTR, including the T7 promoter (T7) and ribosome binding site (RBS), followed by the gene of interest (ATG start codon and the codon-optimized USCTX sequence) and a 35-bp 3′ UTR, including a TAA stop codon and T7 terminator (Ter). Functionally corresponding parts are shown in matching colors, with the UTRs in light gray, the RBS in orange, start/stop codons in dark gray, and promoter/terminator sequences in blue. Spacer nucleotides are indicated by noncontiguous boxes.

The F120 linear gene construct for the production of USCTX consisted of a 49-bp 5′ untranslated region (5′ UTR) including the T7 promoter and ribosome binding site (RBS) separated by nucleotide spacers, the USCTX gene of interest (an ATG start codon and the codon-optimized toxin sequence), and a 35-bp 3′ UTR including a TAA stop codon and the T7 terminator (Figure 1).

### 2.2. Only One Cell-Free System Produces USCTX

**Figure 2:**
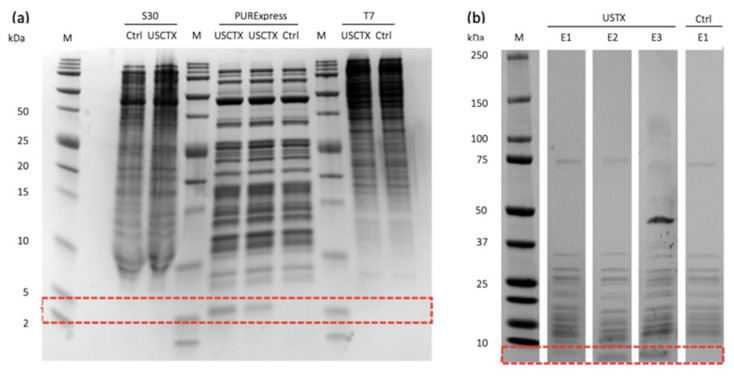
Production and purification of USCTX. (**a**) Commercially available cell-free synthesis systems differ in their ability to produce USCTX (anticipated band size = 4.3 kDa). No such band was produced by the S30 Extract System (left) or the TnT T7 Insect Cell Extract Protein Expression System (right), but a band of the expected size was produced by the NEB PURExpress In Vitro Protein Synthesis System (middle, in duplicate to highlight reproducibility). (**b**) Purification of USCTX, showing the elution fractions E1–E3 from the His-Spin column. The red box indicates the area in which USCTX bands should appear.

F120 was used to assess three commercially available cell-free synthesis systems, two based on the bacterium *Escherichia coli* (PURExpress In Vitro Protein Synthesis System and S30 Extract System) and one based on insects (TnT T7 Insect Cell Extract Protein Expression System). All three were driven by the T7 promoter and were therefore compatible with the F120 construct. The success of each reaction was determined by 1D SDS-PAGE (Figure 2a). The expected size of the USCTX band was 4.3 kDa. No band of that size was produced by the S30 Extract System or the TnT T7 Insect Cell Extract Protein Expression System, whereas a band of the correct size was produced by the NEB PURExpress In Vitro Protein Synthesis System (Figure 2a). The identity of the toxin was confirmed by MALDI-TOF and Orbitrap-based mass spectrometry (see Section 2.5 and Supplementary Figure S1).

To the best of our knowledge, only two previous studies describe the cell-free production of venom proteins. The first used wheat germ lysate to produce isotope-labeled preprosecapin from the venom glands of queen honeybees (*Apis mellifera*), but the activity of the protein was not tested [22]. The second used wheat germ lysate to express snake venom kallikrein, which was found to be almost identical to its natural counterpart in terms of bioactivity [23]. Our study is therefore only the third attempt to use cell-free protein synthesis for the production of venom components and only the second to include bioactivity analysis. The NEB PURExpress In Vitro Protein Synthesis System and wheat germ lysate are therefore the only cell-free systems that have been shown to produce venom components, and the former is the only prokaryote system that has been shown to work thus far. The results of all such studies to date are summarized in Table 1.

Interestingly, USCTX was produced by the NEB PURExpress system but not by the S30 extract even though both are derived from the same bacterial species (*E. coli*). The reasons for this should be explored in more detail to optimize future workflows. The inability of the insect-based TnT T7 system to produce a spider toxin may reflect the codon optimization of the original sequence, which favored *E. coli*. Failure at the level of protein synthesis is more likely than at the level of transcription because all three systems are based on the same T7 promoter and the corresponding transcriptional components are present in the lysates. We compared the codons in F120 to the codon usage preference of *Spodoptera frugiperda*, the source of the insect cells used to prepare the lysate in the TnT T7 system, and we found that all codons in the F120 construct were compatible with insect cells, suggesting that codon preference should not inhibit USCTX synthesis in the TnT T7 system [24]. The failure of two of the cell-free expression systems therefore remains unexplained.

### 2.3. Purification of USCTX

To exclude as many components of the cell-free reaction as possible, we applied a reverse purification strategy based on immobilized metal-ion affinity chromatography (IMAC), as recommended by the manufacturer. This took advantage of the fact that most proteins in the NEB PURExpress In Vitro Protein Synthesis System carry a histidine tag, allowing them to bind the IMAC column while the nontagged USCTX should pass through. However, we found that USCTX did not elute from the column directly and instead required three elution steps (Figure 2b). A small amount of protein was detected in the flow-through fraction of the reaction batch (E1) but more was recovered when the column was eluted with water (E2) and then washing buffer (E3). We therefore collected all three fractions independently for bioactivity screening (Table 2).

### 2.4. USCTX Is Active against Neuroblasts

**Figure 3:**
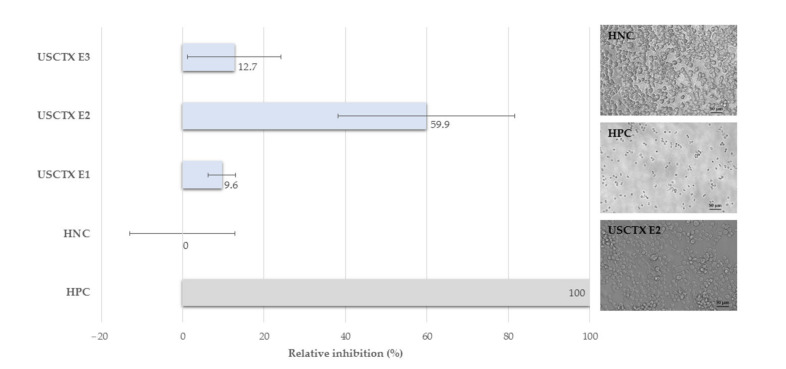
Effect of the recombinant USCTX produced by cell-free expression on the viability of mouse neuroblasts (N2a cells). We tested all three IMAC elution fractions, namely the purification flow-through (E1), water elution (E2), and washing buffer elution (E3). The inhibitory effect was measured relative to the positive control (HPC; maximum inhibition induced by Triton X-100, lowest viability) and negative control (HNC; untreated cells in growth medium, zero inhibition, highest viability).

To determine whether the toxin product retained its bioactivity, we tested each elution fraction against mouse N2a neuroblasts in vitro. This assay was chosen because spider venom ICK proteins are primarily neurotoxic [25]. The assay therefore enabled us to study the effect of toxins on a cell layer enriched with potential targets. Overall, our bioassays revealed a moderate activity of USCTX against murine neuroblasts, with fraction E2 showing the most potent effect (Figure 3).

After correcting for the effect of the pure solvent, fraction E1 achieved only the slight inhibition of N2a cells, reducing their viability by 9.6% ± 3.5%. The effect of fraction E2 was much more potent, reducing the viability of N2a cells by 59.9% ± 21.7%. The effect of fraction E3 was weaker, reducing cell viability by 12.7% ± 11.4%. The bioactivity of each fraction (Figure 3) correlated with the intensity of the USCTX band detected by SDS-PAGE (Figure 2b). Interestingly, the mean effects of E2 and E3 exceeded that of E1 by twofold and tenfold, respectively, even though there did not appear to be a fivefold difference between E2 and E3 in the gel lanes. This suggests that the elution of USCTX with water preserves its activity, whereas the washing buffer may have an inhibitory effect on the toxin.

In terms of cell phenotypes, the toxin appeared to trigger a slight decrease in cell density compared to untreated cells (Figure 3). The cells treated with fraction E2 also showed signs of cytoplasmic membrane disintegration. However, this requires more detailed investigation by cell staining and/or electron microscopy.

### 2.5. Problematic Disulfide Crosslinking in Cell-Free-Expressed USCTX

The production of correctly folded peptides containing an ICK motif is challenging due to the complex structure of the cysteine pseudoknot. Chemical synthesis and heterologous expression in bacteria are particularly laborious because the first method cannot easily control the stereoselective formation of complex disulfide bonds and the second requires specialized expression systems capable of cysteine reduction [26]. An alternative system that allows the rapid production of correctly folded ICK peptides would be a significant advantage.

We assessed the cysteine crosslinking of USCTX by mass spectrometry to determine the efficiency of the cell-free system. The peptide pairs recovered after tryptic digestion reveal disulfide bridges between crosslinked partners that can be identified by their specific masses. Natural USCTX features three disulfide bridges within the ICK involving six cysteines, with C1 connected to C4, C2 to C5, and C3 to C6 [21]. Our mass spectrometry analysis revealed that the expression batch of USCTX was almost exclusively composed of peptide pairs displaying aberrant cysteine crosslinking or peptides that lack disulfide bonds. Only a single peptide with *m*/*z* 1215.56, matching the C2-C5 crosslink of native USCTX, was identified via MALDI and even more sensitive Orbitrap experiments [27,28,29]. Our data suggest that the cell-free system faces difficulties in facilitating the correct disulfide bond formation in such heavily cysteine-crosslinked peptides. The activity of USCTX that we observed therefore probably stems only from those reaction products with incorrect disulfide connectivity. Assessing the bioactivity of native USCTX in future studies and comparing it to our observed activities could help to better understand the impact of cell-free expression-derived aberrant crosslinking on toxin bioactivities.

A potential explanation for the missing and erroneous crosslinks in our USCTX is the missing compartmentalization of cell-free systems. The NEB PURExpress In Vitro Protein Synthesis System is based on the genetic machinery of *E. coli*, which often fails to produce proteins with multiple disulfide bonds. In the cell-free systems, this obstacle is overcome by adding specific enhancers such as protein disulfide isomerases. This essentially converts the original *E. coli* lysate into the equivalent of a lysate from a strain engineered to produce disulfide bridges. However, these engineered cells usually form disulfide bonds in the periplasm, a compartment with an environment that supports the reduction in cysteine residues [30]. The absence of such a compartment in cell-free systems may limit the ability to form disulfide bridges and may thus explain the observed problems in cysteine crosslinking. That said, previous works that employed cell-free systems to produce IgG revealed that these systems can be optimized for their disulfide crosslinking abilities by alterations in disulfide enhancers and their concentrations [31]. Thus, we recommend that, in future works, different disulfide enhancers and other additives should be examined for their capability to produce correct disulfide bonds in USCTX and other animal toxins.

### 2.6. Cell-Free Protein Production for Venom Bioprospecting?

The rapid laboratory-scale production of venom components is advantageous for venom biodiscovery programs because it should enable the bioactivity testing of components from small arthropods. Although the potential offered by cell-free protein synthesis in venomics has been acknowledged in recent studies, there have been few demonstrations thus far [32]. Our work contributes to the expansion of this approach, which is likely to become a key venom biodiscovery strategy in the future. To achieve this goal, it is required to solve several problems that we encountered throughout our examination. The major challenges, explained below in more detail, are the selection of the correct cell-free expression system, the yield–cost–time efficiency, and the correct product folding. The latter challenge is especially pivotal to enable the use of this approach for animal toxin production and assessment, especially for the prominent ICK peptides in arthropod venoms. However, future adjustments to these expression systems may overcome the hurdles in today’s technologies and may establish the cell-free expression as a widespread approach to venom bioprospecting.

In the following, we describe the three major hurdles to cell-free protein expression of arthropod venom ICKs encountered in this study. The first problem that needs to be solved is the selection of an optimal cell-free expression system. As stated above, few systems have been used for venom research despite the availability of diverse cell-free expression systems based on bacteria and eukaryotes. For cell-based expression, the selection of an optimal cell line is a key step in the overall workflow, and a similar strategy may be necessary in cell-free systems to overcome low protein yields and inefficient folding. It may also be necessary to test a variety of cell-free systems derived from the same source organisms because, as our results demonstrated, even the use of similar systems may lead to major differences in protein expression.

The second challenge relates to protein yield. More detailed analysis (including traditional electrophysiology experiments) requires a larger quantity of sample material, but the protein yield in our cell-free batches was very low. However, our experiments used the smallest possible scale, in which only one reaction batch was analyzed. It would be possible to combine several batches in larger vessels to increase the scale. Even so, it is important to point out that upscaling by combining multiple batches may also increase costs, so the economic benefits of cell-free production would need to be reassessed. The small amounts of protein synthesized in cell-free systems could also be isolated and enriched by chromatography, as shown by the micro-fractionation of venom components [33,34,35,36,37]. However, this would also add more experimental steps, thus eliminating one of the major advantages of cell-free expression: the rapid and simple production method.

A final challenge is the prevalence of aberrant cysteine crosslinking. Previous studies of cell-free venom protein expression did not test for correct folding or disulfide crosslinking [22,23]. Our experiments revealed that proteins without disulfide bonds or with incorrect linkages are the only reliably detectable expression products. The ability of the tested cell-free system to produce correct disulfide-crosslinked peptides thus remains questionable. Although the likelihood of erroneous disulfide connectivity is particularly high for small and cysteine-rich peptides, such as USCTX, it is less likely to affect larger proteins with fewer cysteine residues as a proportion of total amino acids (and thus a less complex system of disulfide linkages), which would include most metalloproteinases, CAP family proteins, serine proteases, phospholipases, and hyaluronidases [1,2]. Similarly, cell-free technologies could be employed with linear peptides without disulfide bonds, which are also present in some venoms. We are therefore confident that the selection of an optimal cell-free expression system for venom or a specialized system for certain components will largely solve this problem in the future. In particular, redox conditions, disulfide enhancers, and lower temperatures for slower but more precise protein folding should be considered in this context.

## 3. Conclusions

Cell-free protein production is potentially useful for venom bioprospecting because it allows the rapid, laboratory-scale production of venom components even from small arthropods. Our comparison of three different cell-free production systems by expressing USCTX from the six-eyed sand spider *H. dolichocephala* revealed that only one of the tested systems can produce active USCTX, albeit with small yields and important limitations regarding disulfide bond formation. Despite the theoretical benefits of cell-free production for venom research, the system selection, the yield efficiency, and the protein folding hurdles must be overcome. Given the variable results in different test systems (including the wheat germ lysate used in previous studies), we recommend that future studies should include more diverse prokaryotic and eukaryotic systems, chemical adjustments (e.g., varying disulfide enhancers), as well as a broader set of venom components representing different sizes and varying degrees of cysteine crosslinking. The selection of optimal cell-free systems and their adjustments for each component will facilitate venom bioprospecting in the future and thus provide access to the largely untapped resource of arthropod venoms for basic and translational research.

## 4. Materials and Methods

### 4.1. Sequence Selection and Construct Preparation

The amino acid sequence of USCTX (previously identified in the venom gland transcriptome of *H. dolichocephala*) was obtained from the Arachnoserver database and was codon-optimized for *E. coli* K12 using the EMBOSS package in Geneious [21,38]. We added an N-terminal Glycine residue to slightly increase the protein size and enable better detection in the SDS-PAGE. The complete F120 construct was synthesized by Eurofins Medigenomix (Ebersberg, Germany).

### 4.2. Cell-Free Production and SDS-PAGE

We tested three different commercially available cell-free expression systems: the PURExpress In Vitro Protein Synthesis System (New England Biolabs, Ipswich, MA, USA), the S30 Extract System (Promega, Madison, WI, USA), and the TnT T7 Insect Cell Extract Protein Expression System (Promega) based on the *S. frugiperda* cell line Sf21. We followed the manufacturer’s recommendations for each kit, using 1.5 mL Eppendorf tubes for each reaction. The S30 Extract System lacks methionine, so we added this amino acid to match the concentrations in the other systems. All tubes were stored at −20 °C after the reaction. Protein synthesis was confirmed by 1D SDS-PAGE. We mixed 1 µL of the reaction with 1 µL of Tricine sample buffer and incubated it for 5 min at 95 °C. The sample was then loaded onto a 16.5% Mini-PROTEAN Tris-Tricine Gel (Bio-Rad, Hercules, CA, USA) and placed in a Mini-PROTEAN Tetra System chamber (Bio-Rad) filled with 10x Tris/Tricine/SDS running buffer. After electrophoresis at 100 V for 100 min, the gel was stained overnight with Roti-Blue quick solution (Carl Roth, Karlsruhe, Germany).

### 4.3. IMAC Purification

IMAC chromatography was carried out using the His-Spin Protein Miniprep Kit according to the manufacturer’s instructions (Zymo Research, Irvine, CA, USA). After preparation of the column, the reaction was resuspended in the gel matrix and incubated for 4 min at room temperature before centrifugation at 13,000× g for 10 s. The flow-through was collected as fraction E1. We then added 50 µL of distilled water to the column, followed by centrifugation as above, and the flow-through was collected as fraction E2. Finally, we added 150 µL of the washing buffer supplied in the kit, followed by centrifugation as above, and the flow-through was collected as fraction E3. Purity was assessed by SDS-PAGE. We added 1 µL (E1), 2 µL (E2), or 6 µL (E3) of the elution fractions to an equal volume of Tricine sample buffer and followed the SDS-PAGE and gel staining process described above. Bands corresponding to USCTX were excised and analyzed by mass spectrometry. For subsequent bioassays, elution fractions were lyophilized and redissolved to a volume of 10–13.3% (*v*/*v*) in medium.

### 4.4. MALDI-TOF Mass Spectrometry

Matrix-assisted laser-desorption ionization time-of-flight mass spectrometry (MALDI-TOF MS) was performed on an Ultraflex I TOF/TOF mass spectrometer (Bruker Daltonics, Bremen, Germany) equipped with a nitrogen laser. The instrument was operated in the positive-ion reflectron mode using 2,5-dihydroxybenzoic acid (5 mg/mL; Sigma), and methylenediphosphonic acid (5 mg/mL; Fluka) in 0.1% TFA as matrix. Sum spectra consisting of 200–400 single spectra were acquired. For data processing and instrument control, the Compass 1.4 software package consisting of FlexControl 3.4, FlexAnalysis 3.4, BioTools 3.2 was used. The correlation of the sequence of USCTX with the observed m/z-values was analyzed with BioTools, allowing one missed cleavage. The oxidation and crosslinking of cysteines were set as variable modifications. The obtained MALDI data are given in Supplementary Figure S1.

### 4.5. LC-ESI Mass Spectrometry

For the bottom-up analysis of USCTX, we employed a workflow that was established for the study of animal toxins in the past [39,40]. Briefly, excised gel pieces were destained in 25 mM of ammonium hydrogen carbonate containing 50% (*v*/*v*) acetonitrile and then dehydrated with 100% acetonitrile, reswelled in 50 mM of ammonium hydrogen carbonate, again dehydrated with 100% acetonitrile, and finally dried under vacuum. Free cysteine residues were blocked by incubation with 10 mM of *N*-ethylmaleimide (Sigma-Aldrich, Taufkirchen, Germany) in 50 mM of ammonium hydrogen carbonate. The gel pieces were washed as described above, rehydrated in 30 μL of 25 mM of ammonium hydrogen carbonate containing 10 ng/µL of sequencing grade trypsin (Promega) and 0.025% Proteasemax (Promega), and incubated at 37 °C for 16 h. Peptides were recovered by extraction with 30 μL of 1% trifluoroacetic acid (Applied Biosystems, Warrington, UK) and dried under vacuum. The sample was split into two aliquots. The first, containing disulfide-linked peptides, as well as blocked cysteine residues, was purified using a C18-ZipTip (Merck Millipore, Burlington, MA, USA), dried under vacuum, and redissolved in 10 µL of 0.1% trifluoroacetic acid for direct LC-ESI-MS analysis. The second was treated with 5 mM of DTT (30 min at 50 °C) to break the disulfide crosslinks, and the newly exposed cysteine residues were modified with 10 mM of iodoacetamide (30 min at 24 °C). The reaction was quenched with excess cysteine and the peptides were purified as described above.

For mass spectrometry, we used the highly sensitive Orbitrap instrument on which we previously identified other ICK components [28,29]. Briefly, 0.5 µg of each sample in 0.1% formic acid (Sigma-Aldrich) was loaded onto a 50 cm µPAC C18 column (Pharma Fluidics, Gent, Belgium) mounted on an UltiMate 3000RSLCnano (Thermo Fisher Scientific). The peptides were eluted at 35 °C in a linear gradient of 3–44% acetonitrile over 60 min followed by a wash with 72% acetonitrile at a constant flow rate of 300 nL/min. The peptides were then infused via an Advion TriVersa NanoMate (Advion BioSciences, New York, NY, USA) into an Orbitrap Eclipse Tribrid mass spectrometer (Thermo Fisher Scientific) operating in positive-ionization mode with a NanoMate spray voltage of 1.6 kV and a source temperature at 275 °C. Using data-dependent acquisition mode, the instrument performed full MS scans every 3 s over a mass range of *m*/*z* 375–1500, with the resolution of the Orbitrap set to 120,000. The RF lens was set to 30%, and auto gain control (AGC) was set to standard with a maximum injection time of 50 ms. In each cycle, the most intense ions (charge states 2–7) above a threshold ion count of 50,000 were selected with an isolation window of 1.6 *m*/*z* for higher-energy C-trap dissociation at a normalized collision energy of 30%. Fragment ion spectra were acquired in the linear ion trap with a scan rate set to rapid, mass range set to normal, and a maximum injection time of 100 ms. After fragmentation, the selected precursor ions were excluded for 15 s for further fragmentation.

Data were acquired with Xcalibur v4.3.73.11 and analyzed using Proteome Discoverer v2.5.0.400 (both from Thermo Fisher Scientific). Mascot v2.6.2 (Matrix Science, Boston, MA, USA) was used to search against the USCTX sequence. A precursor ion mass tolerance of 10 ppm was used, and one missed cleavage was allowed. The modification of cysteine residues was optional: carbamidomethylation (former disulfide crosslinks) or alkylation by *N*-ethylmaleimide (free cysteine residues). The oxidation of methionine was also optional. The fragment ion mass tolerance was set to 0.8 Da for the linear ion trap MS^2^ detection. The false discovery rate for peptide identification was limited to 0.01 using a decoy database. The obtained mass spectrometric data are given in Supplementary Table S1.

### 4.6. Bioassays

The effect of USCTX on cell viability was assessed in a fluorescence cell-based bioassay on mouse N2a neuroblasts (ACC148). Viability was expressed by quantifying the ability of cells to reduce resazurin to the fluorescent product resorufin. Briefly, cells were seeded in a 96-well plate at a density of 10,000 cells per well. After 24 h, the cells were treated in three replicates with USCTX in the IMAC elution fractions at concentrations of 10–13.3% (*v*/*v*) in medium. Three replicate wells without cells served as the blank, whereas cells in normal growth medium and cells treated with Triton X-100 (1% *v*/*v*) served as negative and positive treatment controls, respectively. Toxins were incubated with the cells for 24 h before adding a mixture of 1:6 CellTiter Blue reagent containing resazurin (Promega) and fresh medium to each well, followed by incubation for 3 h. Fluorescence readings were acquired on a Synergy H4 hybrid microplate reader (Bio-Tek, Winooski, VT, USA) at wavelengths of 560/590 nm. The influence of USCTX on cell viability was estimated by calculating the percent relative inhibition compared to the positive and negative controls. Raw data are provided in Supplementary Table S2. We classified the relative inhibition value as inactive (<20%), moderately active (20–80%), or active (>80%). The phenotypic effect of USCTX on the cells was documented by observation at 20× magnification using DM IL and DFC425 C inverse microscopes equipped with a five-megapixel camera system (Leica Microsystems, Wetzlar, Germany).

## Figures and Tables

**Table 1:** Summary of the cell-free production of venom components in this study and previous reports, specifying the toxin, source organism, expression system, success of production, and bioactivity testing.

Component	Organism	System	Expression	Activity
Kallikrein [23]	Snake (not determined)	Wheat germ	Yes	Yes
Preprosecapin [22]	*A. mellifera* queens	Wheat germ	Yes	Not tested
USCTX	*H. dolichocephala*	NEB PURExpress (*E. coli*)	Yes	Yes
USCTX	*H. dolichocephala*	S30 Extract (*E. coli*)	No	–
USCTX	*H. dolichocephala*	TnT T7 (*Spodoptera frugiperda*)	No	–

**Table 2:** Characterization of IMAC elution fractions in terms of yield, purification step, and eluent.

Fraction	Toxin Yield	Purification Step	Solvent
E1	Low	Flow-through	Cell-free extract
E2	High	First washing	Water
E3	High	Second washing	Washing buffer

## Data Availability

The data presented in this study are available in the Supplementary Materials.

## Supplementary Materials

The following are available online at https://www.mdpi.com/article/10.3390/toxins13080575/s1, Figure S1: MALDI-spectrum of trypsinized USCTX; Table S1: Raw data from bioassays; Table S2: LC-ESI data.
