# Supplementary material for: A Spider Toxin Exemplifies the Promises and Pitfalls of Cell-Free Protein Production for Venom Biodiscovery
Source: Toxins (Basel). 2021 Aug 18;13(8):575. doi: 10.3390/toxins13080575 (PMC8402385; doi:10.3390/toxins13080575)
Supplement: Supplementary file 1 [file toxins-13-00575-s001.zip › Toxins_1307861_FigureS1.pdf]

toxins

MDPI

Supplementary Materials: A Spider Toxin Exemplifies the Promises and Pitfalls of Cell-Free Protein Production for Venom Biodiscovery

Tim Lüddecke, Anne Paas, Lea Talmann, Kim N. Kirchhoff, Björn M. von Reumont, André Billion, Thomas Timm, Günter Lochnit and Andreas Vilcinskas

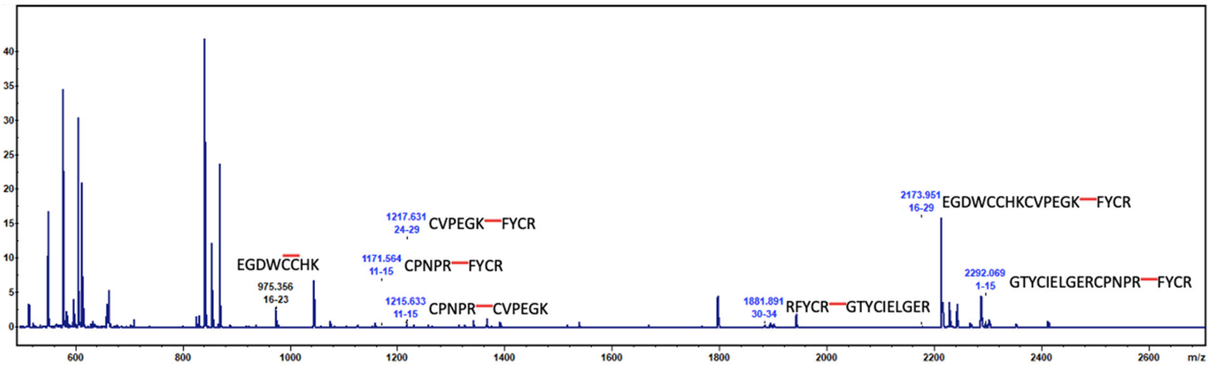

Figure S1. MALDI-spectrum of trypsinized USCTX. The *m*/*z* values corresponding to peptides crosslinked via cysteines are highlighted in blue and are labeled with the crosslinked peptide sequences. The data show a sequence coverage of 94 %, missing only the C-terminal two amino acids (due to a cut off below *m*/*z* 500).
